# The Dutch residency educational climate test: construct and concurrent validation in Spanish language

**DOI:** 10.5116/ijme.5d0c.bff7

**Published:** 2019-07-29

**Authors:** Luis Carlos Dominguez, Milou Silkens, Alvaro Sanabria

**Affiliations:** 1 Department of Surgery, University of La Sabana, Bogotá, Colombia; 2 Professional Performance and Compassionate Care research group, Department of Medical Psychology, Amsterdam UMC, Amsterdam, the Netherlands; 3 Department of Surgery, Universidad de Antioquia, Medellín, Colombia

**Keywords:** Postgraduate medical education, learning climate, psychometrics, D-RECT, Colombia

## Abstract

**Objectives:**

To translate the 35-item version of the Dutch Residency Educational Climate Test (D-RECT), and assess its reliability, construct validity and concurrent validity in the Spanish language.

**Methods:**

For this validation study, the D-RECT was translated using international recommendations. A total of 220 paper-based resident evaluations covering two Colombian universities were cross-sectionally collected in 2015. A Confirmatory Factor Analysis (CFA) was used to assess the internal validity of the instrument using the Comparative fit index (CFI), Tucker-Lewis index (TLI), Standardized root mean square residual (SRMSR), and Root mean square error of approximation (RMSA). Cronbach’s α was used to assess reliability. The concurrent validity was investigated through Pearson correlations with the Spanish version of the Postgraduate Hospital Educational Environment Measure (PHEEM).

**Results:**

The original 9-factor structure showed an appropriate fit for the Spanish version of the instrument (CFI = 0.84, TLI = 0.82, SRMSR = 0.06, and RMSA = 0.06). The reliability coefficients were satisfactory (>0.70). The mean total scores of the D-RECT and the PHEEM showed a significant correlation (r = 0.7, p<0.01).

**Conclusions:**

This study confirms the validity and reliability of the Spanish version of the Dutch Residency Educational Climate Test, indicating that the instrument is suitable for the evaluation of departments’ learning climate in the Spanish context. Future research is needed to confirm these findings in other Spanish speaking countries.

## Introduction

Evaluating the learning climate in postgraduate medical education (PGME) is a cornerstone to assure the quality of residency training as well as of patient care provided by residents. The learning climate can be defined as residents’ perceptions of both formal and informal characteristics of education.^1^^,^^2^ More specifically, the learning climate refers to perceptions about common practices and procedures at the department^3^ as well as the quality of relationships between residents and their clinical teachers, supervisors, and the institution.^4^ Multiple instruments have been developed to evaluate the learning climate in residency training, which exhibit variable grades of validity, reliability, and acceptability.^2^^,^^5^^-^^7^ Despite the increased attention for learning climates in PGME worldwide and the number of instruments that developed as a result of this attention, the availability of such instruments in the Spanish language is limited. This lack of instruments sets boundaries to the continuous improvement of the learning climate in Spanish speaking countries, thereby impacting on academic outcomes that are routinely evaluated in other contexts (e.g., academic performance, self-directed learning, residents’ training experience and wellbeing).^4^^,^^6^^,^^8^^,^^9^

One of the most commonly used instruments to evaluate learning climates in PGME worldwide is the Postgraduate Hospital Educational Environment Measure (PHEEM). The PHEEM was developed through a grounded theory approach involving focus groups and a Delphi method.^2^ A recent systematic review examined the adoption and use of PHEEM in different medical contexts and highlighted its reliability and internal consistency.^10^ For Spanish speaking countries; the PHEEM is the most accepted instrument for the evaluation of the learning climate in PGME.^10^^-^^12^ Another instrument, the Ambulatory Care Learning Education Environment Measure (ACLEEM), is also available in Spanish language but it has received less attention than PHEEM.^13^ ACLEEM is limited to ambulatory settings and involves the evaluation of residents’ support, clinical teaching, and clinical skills. As such, it has been recommended that alternative instruments should be developed and administered in the Spanish-speaking context to provide a more holistic and accurate picture of the educational environment.^11^^,^^14^

As an alternative to the PHEEM, the Dutch Residency Educational Climate Test (D-RECT) was developed in the Netherlands to assess learning climates in PGME.^15^ As opposed to other instruments at the time, the initial 75-item version of the D-RECT was constructed in Dutch and based on a theoretical framework drawn from literature research, qualitative research and a Delphi method.^15^ The instrument was reduced to 50 items in an initial validation study including exploratory, confirmatory, and generalizability analysis, showing appropriate validity and reliability at the resident level. The D-RECT was subsequently translated in English and findings were explored in other settings.^16^^-^^18^ For some settings the psychometric properties of 50-item D-RECT were controversial, requesting a need for further revision.^19^ Simultaneously, the instrument was critically revised in the Netherlands in order to guarantee the validity and reliability on the intended unit of analysis (the clinical departments providing PGME).^20^ This revision showed that the learning climate could be evaluated on both the resident and the department level using a 35-item questionnaire consisting of nine dimensions. This updated version of the D-RECT offered advantages over other instruments aimed at evaluating learning climates in PGME, such as a solid theoretical underpinning, strong psychometric properties due to an extensive validation process, and applicability to the department level.^20^ Although the 35-item D-RECT performed well in the Dutch context, testing its psychometric properties in international contexts and other languages were considered an important knowledge gap.^20^ Up to date, the 35-item D-RECT has only been tested and adjusted to the Filipino context, showing psychometric validity after minor modifications.^21^ This means that there is a need for additional research to further assess the psychometric properties of the 35-items D-RECT in other non-western contexts.

Considering the increasing popularity of the D-RECT, its recent update and the challenges brought forward by the limited number of learning climate instruments in Spanish, this study aimed to translate the 35-item D-RECT into the Spanish language and assess its construct and concurrent validity and reliability. This study aimed to provide psychometric evidence for the D-RECT in the Spanish-speaking context to extend the existing toolkit of instruments available for the evaluation of the learning climate in PGME. This study poses the following research question: what are the construct and concurrent validity, and the reliability of the 35-item D-RECT in the Spanish language?

## Methods

### Study design and participants

The present study was designed as a cross-sectional study that was conducted in two universities in Colombia (one public and one private). In these universities, residents complete full-time training during two to five years (depending on specialty) and subsequently apply to sub-specialties (usually taking two more years). Residents pay a fee to the university but usually do not receive a salary from hospitals, though a few residents receive a semi-annual governmental scholarship. The national directive of duty hours is 66 hours per week.

For the current study, residents from both universities were invited to complete the D-RECT questionnaire. A total of 245 residents from 17 different surgical and non-surgical specialties completed the D-RECT questionnaire. Twenty-five questionnaires were removed from further analysis due to missing data, leaving 220 D-RECT evaluations for further analysis. The mean age of the respondents was 28.8 years (SD = 2.8), of which 120 respondents were female (54.5%), and 97 were male (44.1%). The distribution of respondents according to the postgraduate year of residency (PG-Y) was: PG-Y1 (46.8%), PG-Y2 (26.8%), PG-Y3 (19.1%) and PG-Y4 (6.8%). Table 1 provides further details on the study population.

**Table 1:** Characteristics of the study population (N=220)

Characteristics		Study sample
Age (years), mean (SD)*		28.8 (2.8)
Gender		n (%)
	Male	97 (44.1)
	Female	120 (54.5)
	Missing	3 (1.4)
Year of training		
	1	103 (46.8)
	2	59 (26.8)
	3	42 (19.1)
	4	15 (6.8)
	Missing	1 (0.5)
Specialty		
	Cardiovascular Electrophysiology	2 (0.9)
	Rheumatology	2 (0.9)
	Critical care medicine (paediatrics)	2 (0.9)
	Critical care medicine (adult)	14 (6.4)
	Pulmonary care	4 (1.8)
	Internal medicine	34 (15.5)
	Family medicine	39 (17.7)
	Radiology	10 (4.5)
	General surgery	14 (6.4)
	Anaesthesiology	17 (7.7)
	Physical and rehabilitation medicine	13 (5.9)
	Ophthalmology	4 (1.8)
	Gynaecology	13 (5.9)
	Neurology	4 (1.8)
	Clinical pharmacology	12 (5.5)
	Paediatrics	30 (13.6)
	Orthopaedics	6 (2.7)

*SD = Standard Deviation

### Instruments

We used the D-RECT as well as PHEEM to evaluate the learning climate in PGME. The most recent version of the D-RECT questionnaire is validated in the Netherlands and consists of 35 items covering nine domains (educational atmosphere, teamwork, the role of specialty tutor, coaching and assessment, formal education, resident peer collaboration, work is adapted to residents’ competence, accessibility of supervisors and patient sign-out). 20 Respondents could rate items using a 5-point Likert scale (1 = totally disagree, 2 = disagree, 3 = neutral, 4 = agree, 5 = totally agree).^20^

The validated Spanish PHEEM questionnaire consists of 40 items covering 5 domains (perceptions of teaching and learning, teaching and social support/counselling opportunities, fairness of the program, autonomy and accommodation and catering facilities).^10^ Respondents could rate items using a 5-point Likert scale (1 = totally disagree, 2 = disagree, 3 = neutral, 4 = agree, 5 = totally agree).^10^^,^^12^^,^^22^

An in-depth description of the domains as well as an overview of the conceptual overlap between the two instruments is presented in  Appendix A1.

### Translation of the D-RECT into Spanish

We contacted one of the authors of the original 35-item D-RECT to get authorization for using the questionnaire. We translated the English items into Spanish following the recommendations of the International Quality of Life Assessment project (IQOLA), which were previously applied to translate the SF-36 Health Survey from English into other languages.^23^ Initially, two natively Spanish speaking researchers performed two independent forward translations from English into Spanish (LD, AS). Translations were compared and unified into a single version. Once completed, two certified bilingual translators performed two independent backward translations of from Spanish into English and subsequently, a third certified translator unified these translations into one version. Hereafter, one of the authors of the original 35-item D-RECT compared the resulting backward translation with the original version (MS). To conclude, the main researchers (LD, MS, AS) organized various meetings to clarify any discrepancies and to generate the final Spanish D-RECT. The researchers then conducted a pilot with twenty residents from different specialties in the Faculty of Medicine (Universidad de la Sabana, Colombia) to test the Spanish D-RECT and to appraise whether they found any difficulty, upsetting, or confusing items. Simultaneously the authors assess the process of response. The Spanish version of the D-RECT is available in  Appendix A2.

### Data collection

We prepared a paper-based questionnaire for data collection, which consisted of the Spanish D-RECT, as well as the Spanish PHEEM questionnaire.^10^ We collected data during the weekly meetings of the postgraduate programs in both Colombian universities, during which residents were asked to complete the questionnaire individually. The first author coordinated the admission of the questionnaire during these meetings and was also responsible for the collection of completed questionnaires. Also, the purpose of the study, voluntariness to participate, anonymity, confidentiality and further management of information was explained to residents during these meetings. The data were collected from October to December 2015. Once completed, the first author coordinated the transcription and organization of data in order to guarantee the quality and integrity of information. The ethical approval for the present study was provided by the Commission of Medical Education (Facultad de Medicina, Universidad de la Sabana, Colombia).

**Table 2:** Fit indices for the 9-factor structure of the D-RECT in Spanish and the D-RECT in Dutch

Fit indices	Spanish D-RECT (resident level)	Dutch D-RECT (resident level)	Dutch D-RECT (department level)
CFI	0.84	0.92	0.89
TLI	0.82	0.91	0.88
SRMR	0.06	0.04	0.06
RMSEA	0.06	0.04	0.06

### Data analysis

Frequencies and descriptive statistics were performed to gain insight in relevant study sample characteristics. Resident evaluations that exceeded 50% missing data for the D-RECT or PHEEM were excluded. For both the D-RECT and the PHEEM, subscale scores were computed by averaging item scores. We allowed one item per subscale to have missing data. Similarly, mean total scores were calculated for both instruments. Pearson correlation was used to examine the correlation between the domains as well as mean total scores of both instruments. For the D-RECT data, remaining missing data were imputed using expectation maximization (EM). The internal consistency of the 9-factor structure was assessed by calculating Cronbach’s α. A Cronbach’s α exceeding 0.70 was considered satisfactory.^24^ Corrected item-total correlations were computed to examine the homogeneity of each subscale. An item-total correlation above 0.40 was considered satisfactorily.^25^ Subscales scored satisfactorily on overlap when inter-scale correlations remained under 0.70.

To assess the fit of the 9-factor structure for the Spanish version of the D-RECT, a confirmatory factor analysis (CFA) was performed. Robust maximum likelihood was used to estimate the CFA model. The fit of the model was assessed by combining the following fit indices: the comparative fit index (CFI), the Tucker-Lewis index (TLI), the standardized root mean square residual (SRMR) and the root mean square error of approximation (RMSEA). We used pre-determined cut-off values to assess the fit (CFI and TLI >0.95 for good fit and >0.90 for acceptable fit; SRMR <0.08 for good fit and <0.12 for acceptable fit and RMSEA <0.06 for good fit and <0.10 for acceptable fit).^26^ The Lavaan package in R statistical software version 3.3.1 was used to perform the CFA.^27^ All other analyses were performed in SPSS version 23 (IBM Corp. 2015).

**Table 3:** Pearson correlations for PHEEM and D-RECT domains

D-RECT / PHEEM Domains	Perceptions of teaching and learning	Teaching and social support/counselling opportunities	Fairness of the program	Autonomy	Accommodation and catering facilities
Coaching and assessment	0.61**	0.51**	0.24**	0.68**	0.25**
Accessibility of supervisors	0.52**	0.41**	0.30**	0.55**	0.34**
Role of specialty tutor	0.61**	0.37**	0.29**	0.57**	0.29**
Teamwork	0.43**	0.34**	0.27**	0.40**	0.36**
Resident peer collaboration	0.24**	0.15*	0.13*	0.28**	0.13
Educational atmosphere	0.63**	0.51**	0.35**	0.50**	0.37**
Formal education	0.63**	0.53**	0.35**	0.62**	0.34**
Work is adapted to residents’ competence	0.47**	0.36**	0.27**	0.45**	0.24**
Patient sign-out	0.50**	0.38**	0.18**	0.54**	0.17*

* <0.05; ** <0.01

## Results

### Reliability and construct validity of the D-RECT

Cronbach’s α for the nine subscales of the D-RECT were as follows: 0.78 (subscale ‘work is adapted to residents’ competence’), 0.84 (subscale ‘teamwork’, ‘resident peer collaboration’, and ‘formal education’), 0.86 (subscale ‘educational atmosphere’ and ‘accessibility of supervisors’), and 0.88 (subscale ‘role of specialty tutor’, ‘coaching and assessment’, and ‘patient sign-out’). Corrected item-total correlations ranged from 0.45 (Item “It is possible to do follow up with patients”) to 0.82 (Item “Differences of opinion are not such that they have a negative impact on the work climate”) ( Appendix A2). Inter-scale correlations ranged from 0.21 (subscale ‘patient sign-out’ versus subscale ‘resident peer collaboration’) to 0.76 (subscale ‘coaching and assessment’ versus ‘subscale role specialty tutor’) ( Appendix A3). Table 2 shows the results for the CFA and the original fit indices for the Dutch D-RECT.  Appendix A2 shows the standardized and unstandardized coefficients and the corresponding standard errors for observed and latent variables.

### Concurrent validity of the D-RECT

Mean scores for the subscales of the D-RECT varied from 3.6 (subscale ‘patient sign–out’; SD = 0.9) to 4.3 (subscale ‘resident peer collaboration’; SD = 0.7). Detailed summary statistics for the subscales of the D-RECT are provided in  Appendix A2. The D-RECT had an overall mean score of 3.9 (SD = 0.6). Mean scores for the subscales of the PHEEM varied from 3.3 (subscale ‘teaching and social support/counselling opportunities’; SD=0.6) to 4.0 (subscale ‘teaching and learning’; SD= 0.6). The PHEEM had an overall mean score of 3.8 (SD= 0.5).  Pearson correlation showed all PHEEM and D-RECT domains were significantly correlated, except for the PHEEM domain ‘perceptions of teaching’ and the D-RECT domain ‘resident peer collaboration’ (Table 3). A significant correlation between the overall mean scores of the 35-item D-RECT and the PHEEM (r = 0.7, p = <0.01).

## Discussion

This study translated and validated the revisited 35-item D-RECT questionnaire in the Spanish language by using standard recommendations.^23^ We found that this instrument can evaluate the learning climate in postgraduate training in Spanish with adequate reliability and construct validity. The results obtained from the correlation analysis showed a high level of confidence that the Spanish D-RECT measures a similar construct to the Spanish PHEEM, namely the learning climate in postgraduate training.

The core structure used in the CFA resembles the structure reported by Silkens and colleagues^20^ accounting for nine learning climate domains: educational atmosphere, teamwork, the role of specialty tutor, coaching and assessment, formal education, resident peer collaboration, work is adapted to residents’ competence, accessibility of supervisors and patient sign-out. When investigating the fit indices, the SRMR showed a very good fit and the RMSEA showed good fit, indicating the a priori model fits well to the data. The assumption that the 9-factor structure works well in the Spanish context is further supported by satisfactorily item-total correlations, pointing out that every item contributed to the overall learning climate construct. However, both the CFI and TLI fit indices were <0.9, showing a limited improvement of the tested model to a restricted model. This might suggest that the proposed structure is not the most optimal in the Spanish context. The inter-scale correlations, though mainly very satisfactorily, did show a high correlation between the subscales ‘role of specialty tutor’ and ‘coaching and assessment’. The overlap between these subscales could plead for a merge or textual alteration of the two scales. Overall, the Spanish 35-item D-RECT did satisfactorily meet most of the criteria posed. Especially since reliability coefficients show outstanding performance on all nine subscales, we believe the instrument in its current form is usable in the Spanish context according to previous recommendations.^28^

With regards to the concurrent validity, when comparing the Spanish D-RECT to the Spanish version of the PHEEM, we observed a high correlation between domain scores and total mean scores of both instruments. Only a few studies focused on the concurrent validity of the PHEEM. In a preliminary study, the Job Evaluation Survey Tool (JEST) developed in the UK was correlated against PHEEM, showing high Spearman correlation coefficients.^29^ No previous studies have assessed the concurrent validity of D-RECT, implying the contribution of our study valuable to the validity evidence of the D-RECT. In particular, we found that the positive correlations between the two instruments suggest that the outcomes of instruments are related. Since PHEEM is currently the most often used instrument to assess the learning climate in the Spanish language, a high correlation is promising for the D-RECT, indicating both the PHEEM and D-RECT are multidimensional instruments measuring a similar construct. Nonetheless, there are important differences in the tools’ domains, indicating that both tools are measuring the same construct from different angles. While PHEEM evaluates perceptions of the domains ‘teaching and learning’, ‘social support and counselling’, ‘fairness and inappropriate tasks’, ‘autonomy’, and ‘organization and catering’,^2^^,^^10^  the D-RECT evaluates perceptions of domains such as ‘the educational atmosphere’, ‘teamwork’, ‘role of specialty tutor’ and ‘coaching and assessment’. ^15^^,^^20^ Several domains in both instruments do address similar overarching constructs such as ‘supervision’, ‘social support’ and ‘interaction between professionals at the departments’. However, both instruments address unique domains as well (Table 1). These differences should be taken into account where particular measurements of the learning climate are anticipated, as well as for purposes of comparison between different contexts.

### Strengths and limitations of the study

The present study adds support for the 35-item D-RECT in a new context, namely the Spanish language. We recruited medical residents from different universities, including public and private institutions, with a wide distribution of specialties that are representative of common conditions in Latin America, guaranteeing the generalizability of the results. Furthermore, we followed a rigorous process of translation following international recommendations and pilot tested the Spanish items in a small group of residents before the start of data collection.

The main limitation of the present study is that the D-RECT was tested in a non-aggregated form, providing information on the resident level only. As these limitations are due to the sample size achieved in the study, researching a wider number of residency programs would support the generalizability of results to the department level. Furthermore, a larger sample could offer information about differences between programs: information the present study is not able to provide. Another limitation of the present study is the absence of exploratory factor analysis. Following the example of the original validation of the D-RECT, we preferred splitting the sample and used part of the sample for exploratory factor analysis and the other part of the sample to confirm the identified structure with a CFA. However, as the original validation procedure of the D-RECT already identified a solid structure by using a CFA and as the content of the D-RECT was applicable in the Colombian context without alterations, we identified a CFA as the preferred method to assess the psychometric properties of the Spanish instrument. Finally, although the results of the present study provide a Spanish version of the D-RECT that is valid and reliable, other Spanish speaking countries should thoroughly assess the instrument to decide whether the instrument applies to other Spanish contexts in terms of content.

### Implications for practice and future research

This study contributes to the lack of instruments for evaluating the learning climate in the Spanish language. The 35-item D-RECT can now be added to the toolbox of valid learning climate instruments in residency training. The validation of this instrument offers a standardized and objective method to evaluate the learning climate in Spanish language countries and to compare its different educational contexts, for example, compare differences in performance between specialties. Furthermore, it provides a reliable tool to evaluate improvement after curricular and educational changes are done.^3^^,^^30^^-^^32^ Ultimately, the instrument can be used for research into the effect of the learning climate on patient outcomes and health care system cost-effectiveness. A good instrument to measure the learning climate in residency training offers an opportunity to understand the internal elements of climate and its relationship with patient outcomes.

When educators and researchers wish to evaluate the learning climate, it is important that they assess their goals in order to pick the right instrument from the existed toolbox. Whether the PHEEM or the D-RECT is the preferred instrument might depend on the goals of evaluation, and as such, cautiousness is advised for proper selection. Above all, it is important to realize that instruments such as the PHEEM and the D-RECT need continuous attention. Similarly, further studies are needed to establish the concurrent validity of the Spanish versions of D-RECT and PHEEM against, for example, ACLEEM.^13^ The practice is changing all the time, and so should these instruments to adapt to these changes. As we stated above, the D-RECT should be tested in other Spanish speaking countries were conditions for residency training, and wellbeing during training could be affected by different health and educative systems. We call for further research in this area.

## Conclusions

In conclusion, our study demonstrates that the Spanish version of the 35-item D-RECT is valid, reliable, and a contextually appropriate instrument for the evaluation of the learning climate of postgraduate training in Spanish language contexts. The use of this instrument contributes to the toolkit of available instruments for measurement of learning climate, such as the PHEEM, in order to improve the quality of postgraduate education.

### Conflict of Interest

The authors declare that they have no conflict of interest.

## Supplementary material

Supplementary file 1 Appendix A1. Content of domains for the PHEEM and D-RECT as well as conceptual overlap

Supplementary file 2 Appendix A2. Standardized and unstandardized coefficients with standard error, corrected item-total correlations, reliability coefficients, and summary statistics for the Spanish D-RECT

Supplementary file 3 Appendix A3. Inter-scale correlations
