# Supplementary material for: The Dutch residency educational climate test: construct and concurrent validation in Spanish language
Source: Int J Med Educ. 2019 Jul 29;10:138–48. doi: 10.5116/ijme.5d0c.bff7 (PMC6773368; doi:10.5116/ijme.5d0c.bff7)
Supplement: Supplementary file 1 — Appendix A1. Content of domains for the PHEEM and D-RECT as well as conceptual overlap [file ijme-10-138-S1.pdf]

Appendix A1

Content of domains for the PHEEM and D-RECT as well as conceptual overlap

Overarching themes	PHEEM		D-RECT	
	Domains	Content of Items	Domains	Content of Items
Supervision	Perceptions of teaching and learning	Arrangement of learning opportunities and aspects of supervision during learning opportunities and providing patient care	Coaching and assessment	Aspects of supervision and feedback behaviour of supervisors
			Accessibility of supervisors	The availability of supervisors when residents are in need
Social support and overall guidance	Teaching and social support/ counselling opportunities	Available time, social support, and resources for residents to participate in educational and career activities as well as patient care	Role of specialty tutor	Involvement of the specialty tutor in the learning trajectory of the resident
			Teamwork	Collaborating relations with other healthcare staff at the department
			Resident peer collaboration	Collaborating relations amongst residents
Appropriate interaction	Fairness of the program	The occurrence of inappropriate behaviour and exploitation of residents	Educational atmosphere	The atmosphere at the department, the occurrence of conflicts and the way in which people treat each other
Clarity of information	Autonomy	Clarity of program information provided	-	-
Quality of work environment	Accommodation and catering facilities	Quality of accommodation and catering	-	-
Quality of formal education	-	-	Formal education	The quality and continuity of planned educational moments for residents
The balance between work and knowledge/ skills	-	-	Work is adapted to residents' competence	The suitability of the tasks provided to the resident relatively to the knowledge and skills base of the resident
Patient sign-out	-	-	Patient sign-out	The degree in which patient sign-outs are used as a moment to educate residents
