# Supplementary material for: The Dutch residency educational climate test: construct and concurrent validation in Spanish language
Source: Int J Med Educ. 2019 Jul 29;10:138–48. doi: 10.5116/ijme.5d0c.bff7 (PMC6773368; doi:10.5116/ijme.5d0c.bff7)
Supplement: Supplementary file 2 — Appendix A2. Standardized and unstandardized coefficients with standard error, corrected item-total correlations, reliability coefficients, and summary statistics for the Spanish D-RECT [file ijme-10-138-S2.pdf]

Appendix A2

Standardized and unstandardized coefficients with standard error, corrected item-total correlations, reliability coefficients, and summary statistics for the Spanish D-RECT

Scale	Item	β/B(SE)*	Corrected item-total correlation	Cronbach's alpha	Mean (SD)
Educational atmosphere	Continuity of care is not affected by differences of opinion between attendings.<br>*(La continuidad de la atención no es afectada por las diferencias de opinión de los especialistas)*	0.72/1.00 (-)	0.659	0.858	3.7 (0.9)
	Differences of opinion between attendings about patient management are discussed in such a manner that is instructive to others present.<br>*(Las diferencias de opinión de los especialistas sobre el manejo del paciente se discuten de una manera que es instructiva para las demás personas presentes)*	0.71/0.89 (0.08)	0.619		
	Differences of opinion are not such that they have a negative impact on the work climate.<br>*(Las diferencias de opinión no tienen un efecto negativo en el clima de trabajo)*	0.87/1.26 (0.10)	0.824		
	There is (are) NO attending physician(s) who have a negative impact on the educational climate.<br>*(No hay ningún especialista que tenga un efecto negativo en el clima educativo)*	0.78/1.19 (0.09)	0.712		
	My attendings treat me with respect.<br>*(Los especialistas me tratan con respeto)*	0.67/0.72 (0.09)	0.574		
Teamwork	Attendings, nursing staff, other allied health professionals and residents work together as a team.<br>*(Los especialistas, el personal de enfermería, el resto del personal de atención médica y los residentes trabajan en equipo)*	0.78/1.00 (-)	0.654	0.840	3.9 (0.8)
	Nursing staff and other allied health professionals make a positive contribution to my training.<br>*(El personal de enfermería y el resto del personal de atención médica contribuyen positivamente a mi entrenamiento)*	0.87/1.02 (0.06)	0.787		
	Nursing staff and other allied health professionals are willing to reflect with me on the delivery of patient care.<br>*(El personal de enfermería y resto del personal de atención médica están dispuestos a reflexionar conmigo sobre como se lleva a cabo el cuidado del paciente)*	0.78/1.01 (0.07)	0.682		
Role of specialty tutor	The specialty tutor monitors the progress of my training.<br>*(El coordinador de la especialización supervisa el progreso de mi entrenamiento)*	0.82/1.00 (-)	0.744	0.880	3.8 (0.8)
	The specialty tutor provides guidance to other attendings when needed.<br>*(El coordinador de la especialización orienta a otros especialistas cuando es necesario)*	0.81/0.96 (0.05)	0.737		
	The specialty tutor is actively involved in improving the quality of education and training.<br>*(El coordinador de la especialización participa activamente en el mejoramiento de la calidad de la educación y el entrenamiento)*	0.79/0.87 (0.06)	0.737		

	In this rotation evaluations are useful discussions about my performance. *(En esta rotación, las evaluaciones son discusiones útiles sobre mi desempeño)*	0.75/0.83 (0.07)	0.721		
	My plans for the future are part of the discussion. *(Mis planes de cara al futuro forman parte de la discusión)*	0.61/0.74 (0.08)	0.552		
	During evaluations, input from several attendings is considered. *(Durante las evaluaciones se toman en cuenta los puntos de vista de varios especialistas)*	0.71/0.76 (0.08)	0.652		
Coaching and assessment	My attendings take the initiative to evaluate my performance. *(Los especialistas toman la iniciativa para evaluar mi desempeño)*	0.79/1.00 (-)	0.761	0.882	3.7 (0.8)
	My attendings take the initiative to evaluate difficult situations I have been involved in. *(Los especialistas toman la iniciativa para evaluar las situaciones difíciles que he tenido que enfrentar)*	0.78/1.07 (0.08)	0.725		
	My attendings evaluate whether my performance in patient care is commensurate with my level of training *(Los especialistas evalúan si mi desempeño en la atención del paciente es apropiado para mi nivel de entrenamiento)*	0.81/0.92 (0.07)	0.734		
	My attendings occasionally observe me taking a history. *(Los especialistas ocasionalmente me observan realizando una historia clínica)*	0.55/0.81 (0.11)	0.525		
	My attendings assess not only my medical expertise but also other skills such as teamwork, organization or professional behavior. *(Los especialistas evalúan no solo mis conocimientos médicos, sino también otras aptitudes como el trabajo en equipo, mi organización o mi conducta profesional)*	0.81/0.98 (0.09)	0.726		
	My attendings give regular feedback on my strengths and weaknesses *(Los especialistas me retroalimentan frecuentemente sobre mis fortalezas y debilidades)*	0.79/1.21 (0.10)	0.737		
Formal education	Residents are generally able to attend scheduled educational activities. *(Los residentes en general pueden asistir a las actividades educativas programadas)*	0.61/1.00 (-)	0.604	0.840	3.8 (0.8)
	Educational activities take place as scheduled. *(Las actividades educativas se llevan a cabo según lo programado)*	0.64/1.02 (0.10)	0.636		
	Attendings contribute actively to the delivery of high-quality formal education. *(Los especialistas contribuyen activamente al logro de una educación formal de alta calidad)*	0.90/1.32 (0.13)	0.750		
	Formal education and training activities are appropriate to my needs. *(La educación formal y las actividades de entrenamiento satisfacen mis necesidades)*	0.86/1.34 (0.16)	0.712		
Resident peer collaboration	Residents work well together. *(Los residentes trabajan bien entre ellos)*	0.85/1.00 (-)	0.731	0.843	4.3 (0.7)
	Residents, as a group, make sure the day's work gets done.	0.80/0.83 (0.10)	0.724		

	(Como grupo, los residentes se encargan de que se realice el trabajo diario)				
	Within our group of residents, it is easy to find someone to cover or exchange a call. *(En nuestro grupo de residentes, es fácil encontrar a alguien con quien intercambiar o cubrir alguna actividad)*	0.77/0.95 (0.06)	0.684		
Work is adapted to residents' competence	The work I am doing is commensurate with my level of experience. *(El trabajo que hago es apropiado para mi nivel de experiencia)*	0.88/1.00 (-)	0.743	0.781	4.0 (0.7)
	The work I am doing suits my learning objectives at this stage of my training. *(El trabajo que hago se adecua a mis objetivos de aprendizaje en esta etapa entrenamiento)*	0.87/1.00 (0.09)	0.691		
	It is possible to do follow up with patients. *(Es posible hacer seguimiento del estado de salud de los pacientes)*	0.52/0.59 (0.10)	0.446		
Accessibility of supervisors	When I need an attending, I can always contact one. *(Cuando necesito a un especialista, siempre puedo ponerme en contacto con uno)*	0.85/1.00 (-)	0.751	0.864	4.0 (0.7)
	When I need to consult an attending, they are readily available. *(Cuando necesito consultar algo con un especialista, lo encuentro disponible rápidamente)*	0.87/1.05 (0.07)	0.794		
	It is clear which attending supervises me. *(Está claro qué especialista me supervisa)*	0.79/1.10 (0.09)	0.698		
Patient sign-out	Sign-out is used as a teaching opportunity. *(La entrega de información del paciente durante el cambio de turno (guardia) se utiliza como oportunidad de enseñanza)*	0.84/1.00 (-)	0.782	0.878	3.6 (0.9)
	Attendings encourage residents to join in the discussion during sign-out. *(Los especialistas fomentan que los residentes participen en la discusión durante el cambio de turno (guardia)*	0.93/1.12 (0.07)	0.782		

*β = standardized coefficients; B = unstandardized coefficients; SE = standard error
