# Supplementary material for: The Dutch residency educational climate test: construct and concurrent validation in Spanish language
Source: Int J Med Educ. 2019 Jul 29;10:138–48. doi: 10.5116/ijme.5d0c.bff7 (PMC6773368; doi:10.5116/ijme.5d0c.bff7)
Supplement: Supplementary file 3 — Appendix A3. Inter-scale correlations [file ijme-10-138-S3.pdf]

Appendix A3

Inter-scale correlations

Domains	Educational atmosphere	Teamwork	Role of specialty tutor	Coaching and assessment	Formal education	Resident peer collaboration	Work is adapted to residents' competence	Accessibility of supervisors	Patient sign-out
Educational atmosphere	1	0.64	0.65	0.60	0.66	0.36	0.50	0.53	0.49
Teamwork	-	1	0.58	0.54	0.51	0.53	0.56	0.54	0.36
Role of specialty tutor	-	-	1	0.76	0.69	0.45	0.58	0.62	0.57
Coaching and assessment	-	-	-	1	0.68	0.38	0.58	0.59	0.57
Formal education	-	-	-	-	1	0.48	0.57	0.60	0.51
Resident peer collaboration	-	-	-	-	-	1	0.53	0.52	0.21
Work is adapted to residents' competence	-	-	-	-	-	-	1	0.65	0.57
Accessibility of supervisors	-	-	-	-	-	-	-	1	0.46
Patient sign-out	-	-	-	-	-	-	-	-	1
